# Olfactory Delusional Syndrome and Intracranial Meningioma

**DOI:** 10.1155/2011/395106

**Published:** 2011-05-30

**Authors:** Michele Rotondo, Massimo Natale, Raffaele D'Avanzo, Assunta Scuotto

**Affiliations:** ^1^Department of Neuroscience, Second University of Naples, 81100 Caserta, Italy; ^2^CTO Hospital, Second University of Naples, Colli Aminei Avenue, 2180121 Naples, Italy

## Abstract

We report the case of a 37-year-old female in which the removal of a suprasellar neoplasm was accompanied by the disappearance of a longstanding olfactory delusion syndrome. In primary care the patient condition was exclusively thought to be psychic in origin, neglecting the possible, not infrequent, organic contribution. The delayed diagnosis produced neurological impairment, only partially recovered after surgical therapy. This case might help to improve the patient management via multi-specialist cooperation and to broaden the knowledge about somatic mechanisms of psychic disturbances, are not often taken into account.

## 1. Introduction

It is well documented that organic factors (intracranial neoplasm, cerebral hypoperfusion, head trauma, hyponatremia, hypothyroidism, etc.) can produce psychic diseases [1–4], including olfactory delusional syndrome (ODS). However, in most cases this circumstance is recognized only retrospectively, thus delaying the diagnosis and compromising the outcome as the case of our patient. The estimated incidence of an organic etiology of psychic diseases remains still now vague but—undeniably—it appears not unusual as in the past. In fact, a sharp increase in the number of relevant published cases has occurred in the last years [3, 5].

## 2. Case Report

37-year-old female presented to medical department with one-week history of headache, weakness both legs, memory loss, insomnia, and firm belief that she was continuously emitting a foul odor from her mouth. She was diagnosed with psychic disease, thus addressed to psychiatrist, and consequently treated for olfactory delusional syndrome. 

One year later she presented to our institution because of unbearable frontal headaches, blurred vision, and persistent preoccupation with fetor ex ore. 

On admission neurological examination revealed bilateral Babinski sign, mild paraparesis, and decrease of visual acuity. There was no anosmia. She denied ever having constitutional symptoms or a history of intravenous drug use. There were no significant baseline laboratory abnormalities. No medical condition was found to explain her beliefs about body odors. 

**Figure 1:**
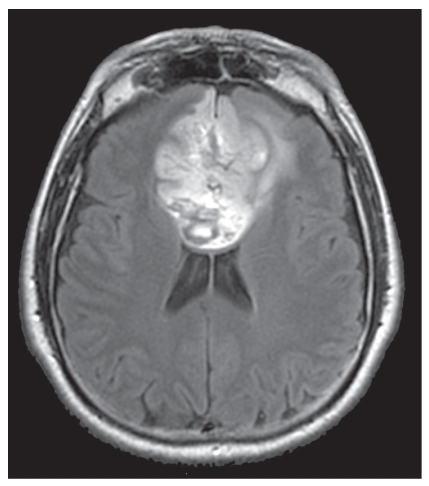
Axial FLAIR MR shows a large inhomogeneously hyperintense, extraaxial mass, located in the frontobasal region. The lesion compresses and displaces both the frontal horns of lateral ventricles and the corpus callosum.

She subsequently underwent magnetic resonance imaging (MRI) of the brain, that showed a frontobasal suprasellar large neoplasm with compression of corpus callosum and paracentral lobes (Figure 1). The presence of a meningioma was suspected and ultimately confirmed by histology after surgical removal. The total excision of the mass via bicoronal approach was followed by the disappearance of both psychic disturbances and paraparesis. The visual impairment—although improved—is still present (6 months follow up).

## 3. Discussion and Conclusion

A delusional disorder is a condition where the person develops an insidious onset of firm, unshakeable, false belief, whose central theme may be persecutory, grandiose, jealousy, erotic, somatic, or mixed. Often the main topic in somatic delusional disorder is that of the conviction that the individual emits a foul odor from the mouth, skin, or genital. 

Although often described in relation to psychotic states, it is, nevertheless, widely considered that this sort of syndrome might have an anatomical basis because of their frequent association with organic brain disease [6, 7]. In the presented case, the halitosis complaint was presented as patient main concern. In fact, she complained the fear of emitting body odor, the idea that the smell made people around her disgusted, and the consequent conviction that she was disliked. Her embarrassment about the perceived foul odor gradually caused increasing social/working withdrawal and isolation. On this basis, in primary care an ODS was diagnosed. However, the cohort of symptoms as persistent headache and weakness both legs should have alerted to investigate the existence of possible organic contributions. 

At this regard, it is mandatory and contemporary easy to ascertain

- drug and/or medication abuse,
- general medical condition (e.g., etc. hypothyroidism, hyponatremia, etc.),
- neurological history (i.e., head trauma, etc.); furthermore, neurological examination is needed for detecting any sign—if present—of nervous system involvement (i.e., Babinski sign in the reported case); it would be always performed in patients with psychic disorders especially when the syndrome long lasts and appears not responsive to the specific treatment.

If clinical features as well as history are suspicious for organic brain disease, MR would be warranted to detect brain involvement and to promptly allow the appropriate therapeutic strategy. 

In the reported case, MR disclosed a large suprasellar neoplasm. Its surgical removal was accompanied by disappearance both of ODS and of paraparesis. 

The mechanism of ODS appears to have been the result of reversible organic damage may be involving mainly the hippocampus [8, 9]. As suggested by Takeuchi et al. [10], who firstly reported in 1993 an ODS due to an intracranial suprasellar mass, the symptoms may have resulted from the stimulation of the olfactory pathways, such as the olfactory tract, olfactory trigone, amygdala, and limbic system by the neoplasm along with compression of the subcallosal area. As well known, anatomical studies have confirmed the view that the hippocampal formation receives a strong olfactory input. It is also well established that the entorhinal cortex receives a direct input from the olfactory bulb and, further, that some individual entorhinal neurons that project to the hippocampus receive a direct input from the olfactory bulb. 

The main factors contributing to misdiagnosis could be identified as

- the failure to consider an organic cause of a psychic disease,
- the negligence to submit the patient to neurological examination and, consequently, neuroradiological investigation.

The final diagnosis was reached after the visual impairment occurred and the headache became permanent, the clinical examination was performed, and MRI was carried out. 

The delayed diagnosis produced visual deficit, still now present, along with a longstanding significant distress in occupational and social functioning. Moreover, the delayed diagnosis put off the proper surgical therapy, always indicated when intracranial mass is found in order to remove the cause of severe neurological deficit and eventually exitus.
